# Quantitative Proteomic Analysis of Zearalenone Exposure on Uterine Development in Weaned Gilts

**DOI:** 10.3390/toxins14100692

**Published:** 2022-10-09

**Authors:** Xinglin Liu, Zengchun Wang, Yanping Jiang, Libo Huang, Xuejun Yuan, Yang Li, Ning Jiao, Weiren Yang, Shuzhen Jiang

**Affiliations:** 1 Colloge of Animal Sciences and Veterinary Medicine, Shandong Agricultural University, No. 61 Daizong Street, Tai’an 271018, China; 2 Shandong Muge Animal Husbandry Equipment Co., Ltd., Yucheng 251200, China; 3 Zhongcheng Feed Technology Co., Ltd., Feicheng 271600, China; 4 Department of Life Sciences, Shandong Agricultural University, No. 61 Daizong Street, Tai’an 271018, China

**Keywords:** zearalenone, uterine development, gilts, serum hormones, proteomic

## Abstract

The aim of this study was to explore the effect of zearalenone (ZEA) exposure on uterine development in weaned gilts by quantitative proteome analysis with tandem mass spectrometry tags (TMT). A total of 16 healthy weaned gilts were randomly divided into control (basal diet) and ZEA3.0 treatments groups (basal diet supplemented with 3.0 mg/kg ZEA). Results showed that vulva size and uterine development index were increased (*p* < 0.05), whereas serum follicle stimulation hormone, luteinizing hormone and gonadotropin-releasing hormone were decreased in gilts fed the ZEA diet (*p* < 0.05). ZEA, α-zearalenol (α-ZOL) and β-zearalenol (β-ZOL) were detected in the uteri of gilts fed a 3.0 mg/kg ZEA diet (*p* < 0.05). The relative protein expression levels of creatine kinase M-type (CKM), atriopeptidase (MME) and myeloperoxidase (MPO) were up-regulated (*p* < 0.05), whereas aldehyde dehydrogenase 1 family member (ALDH1A2), secretogranin-1 (CHGB) and SURP and G-patch domain containing 1 (SUGP1) were down-regulated (*p* < 0.05) in the ZEA3.0 group by western blot, which indicated that the proteomics data were dependable. In addition, the functions of differentially expressed proteins (DEPs) mainly involved the cellular process, biological regulation and metabolic process in the biological process category. Some important signaling pathways were changed in the ZEA3.0 group, such as extracellular matrix (ECM)-receptor interaction, focal adhesion and the phosphoinositide 3-kinase–protein kinase B (PI3K-AKT) signaling pathway (*p* < 0.01). This study sheds new light on the molecular mechanism of ZEA in the uterine development of gilts.

## 1. Introduction

Zearalenone (ZEA) is a mycotoxin mainly produced by *Fusarium fungi*, extensively exposed in corn, wheat and other grain crops [1]. Similar to natural estrogens in structure, ZEA can bind to classical estrogen receptors (ERα and ERβ) and non-classical estrogen receptors (GPER), which disrupts hormone balance and induces reproductive diseases [2,3]. Therefore, ZEA can lead to clinical diseases such as uterine hypertrophy, vulva swelling, reproductive tract prolapses and infertility [4,5]. Moreover, ZEA has shown severe cytotoxicity, which can destroy mitochondrial structure and induce oxidative stress and cell apoptosis via producing reactive oxygen species (ROS) [2,6,7]. Prepubertal gilts are especially sensitive to ZEA [8]. A study reported that 20 and 40 μg/kg body weight (BW) ZEA could induce uterine swelling of prepubertal gilts [9]. Our previous studies showed that uterine size of gilts was increased under ZEA (more than 1 mg/kg) treatment [10,11]. In addition, the relative mRNA and protein expressions of proliferating cell nuclear antigen (PCNA), BCL-2 associated X protein (BAX), and B-cell lymphoma/leukemia-2 (BCL-2) were up-regulated through the TGF-β1/Smad3 signaling pathway in uteri of gilts fed 1.5 mg/kg of a ZEA-contaminated diet [12]. Changes of relative mRNA and protein expressions of GSK-3 and Cyclin D1 of the Wnt/β-catenin signaling pathway in ZEA-treated porcine endometrial epithelial cells were also observed [13]. However, the potential physiological differences and certain molecular mechanisms of ZEA-induced uterine hypertrophy are unclear.

The gene and mRNA levels did not absolutely reflect the physiological changes of organisms due to pre- or post-transcriptional regulation [14]. Proteomics technologies are powerful tools to evaluate the complex biological functions of organisms and analyze the complete protein composition by post-transcription [15]. Quantitative proteome analysis with tandem mass spectrometry tags (TMT) is accurate and stable, and is widely used to identify changes in protein expression levels under physiological or pathological conditions [16,17]. To date, many studies have revealed the molecular mechanisms of diverse tissues or cells treated with ZEA based on proteomics technologies [7,18,19,20]. However, the proteomic changes of uterine development in gilts fed with 3.0 mg/kg of ZEA have not been reported.

The aim of our study was to identify the changes of global protein expression and important signaling pathways through TMT proteomics technologies in uteri of gilts fed with 3.0 mg/kg of ZEA. The serum hormone level and zearalenone and its metabolites’ content were also evaluated.

## 2. Results

### 2.1. Growth Performance

After 32 days of feeding, there was no significant difference in final BW, average daily feed intake (ADFI), average daily gain (ADG), and feed/gain (F/G) between the two groups (*p* > 0.05, Table 1).

### 2.2. Vulva Size

The final vulva area, final area/initial area, and final area/final weight of gilts fed with 3.0 mg/kg of ZEA diet were higher than those of gilts fed with control diet (*p* < 0.05, Table 2).

### 2.3. Serum Hormone

As shown in Table 3, there was no significant difference in serum estradiol and progesterone between the two groups (*p* > 0.05), whereas serum follicle stimulation hormone, luteinizing hormone, and gonadotropin-releasing hormone of ZEA-treated gilts were lower than those of control gilts (*p* < 0.05).

### 2.4. Content of Zearalenone and Its Metabolites in the Uterus

As shown in Table 4, ZEA and its metabolites α-zearalenol (α-ZOL) and β-zearalenol (β-ZOL) contents were not detected or were lower than the detection limit in the uteri of gilts fed with control diet. However, ZEA, α-ZOL and β-ZOL were all detected in the uteri of gilts fed with 3.0 mg/kg of ZEA diet, at levels higher than those of gilts fed with control diet (*p* < 0.05).

### 2.5. Uterine Development Index

**Figure 1:**
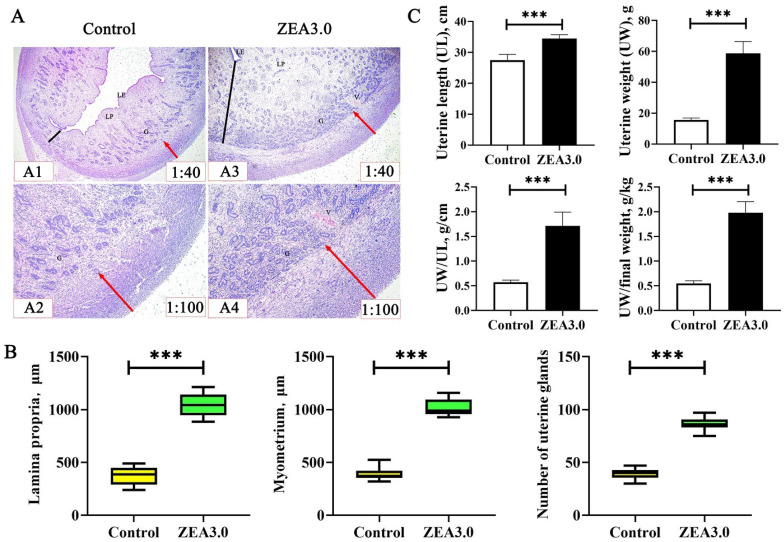
Effects of zearalenone (ZEA) on uterine development index of weaned gilts (*n* = 8). (**A**) Uterine development photomicrographs. (**B**) Thickness of lamina propria and myometrium, and number of uterine glands. (**C**) Uterine development index. The black straight lines indicate the thickness of the lamina propria (LP). The red straight arrows indicate the thickness of the myometrium. The G and V are uterine gland and vessel, respectively. The 1:40 and 1:100 represent the view of uterine samples at 40 magnification and 100 magnification, respectively. Data are mean value ± standard deviation (*n* = 8). *** Means differ significantly (*p* < 0.001).

The uterine development results of weaned gilts were shown in Figure 1. Intuitively, the lamina propria (the black straight lines, A1 and A3) and muscularis (the red straight arrows, A1, A2, A3, and A4) in ZEA 3.0 group were thicker than those in the control group, and the number of glands (G) was more than that in the control. Statistical analysis showed that the thickness of lamina propria and myometrium and the number of uterus gland of the ZEA 3.0 group were significantly increased (*p* < 0.05) compared with the control group (B). Meanwhile, the uterine length (UL), uterine weight (UW), UW/UL and UW/final weight of gilts in the ZEA 3.0 group were significantly increased compared with those in the control group (*p* < 0.05, C).

### 2.6. Sodium Dodecyl Sulfate PolyAcrylamide Gel Electrophoresis (SDS-PAGE) and Differentially Expressed Proteins (DEPs)

**Figure 2:**
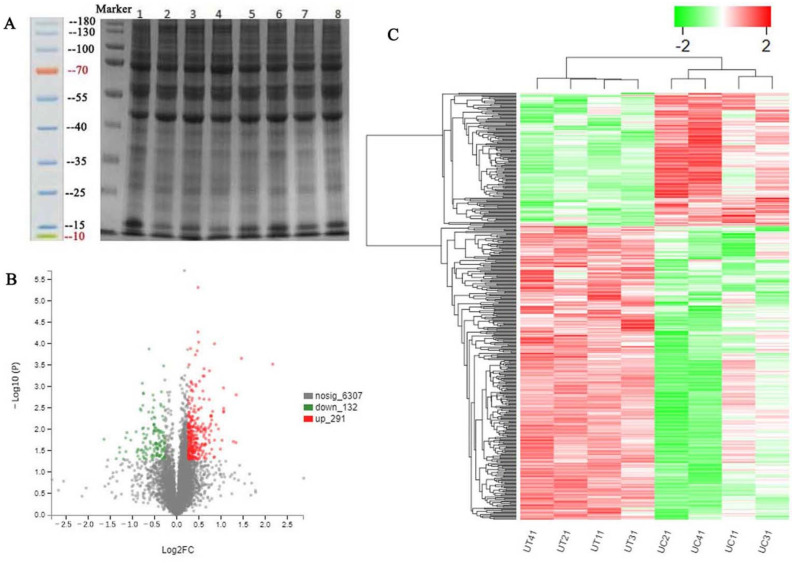
Differentially expressed proteins (DEPs). (**A**) The SDS-PAGE gel electrophoresis profile of uterine proteins in gilts. (**B**) The volcano map of DEPs. (**C**) Hierarchical cluster heatmap analysis of DEPs between control and ZEA3.0 treatment group. The red and green dots or strips represent up-regulated and down-regulated DEPs, respectively. UC11, UC21, UC31, and UC41 represent uterine samples from the control group, and UT11, UT21, UT31, and UT41 represent uterine samples from the ZEA3.0 treatment group.

Proteomic analysis was performed to further explore the signaling pathway of uterine hypertrophy caused by ZEA treatment. The results of SDS-PAGE showed that the sample bands were rich and clear in the control and ZEA3.0 treatment groups (Figure 2A). A total of 6730 proteins were identified via liquid chromatography–tandem mass spectrometry (LC-MS/MS) from the ZEA 3.0 and control groups (Table S1). Compared to the control group, there were 423 significant DEPs in the ZEA 3.0 group; the up-regulated and down-regulated DEPs numbered 291 and 132 in the ZEA 3.0 group, respectively (Figure 2B, Table S2). To analyze the difference between these two groups, they are displayed in the form of a hierarchical clustering heat map (Figure 2C, Table S3). The red and green strips represent the up-regulated DEPs (FC > 1.2, *p* < 0.05) and down-regulated DEPs (FC < 0.83, *p* < 0.05), respectively.

The top 10 up-regulated and down-regulated proteins are shown in Table 5. The up-regulated proteins in the ZEA 3.0 group were energy metabolism-related proteins such as Q5XLD3 (CKM), unique E3 ubiquitin ligase activity protein F1RLE9 (TRIM36), autophagy related protein A0A4X1UF72, neutral endopeptidase A0A287BPD6 (MME), and cell growth and motility proteins A0A5G2R354 (SAXO2), but the function of up-regulated protein A0A4X1THU1 was not clear. Compared with the control group, proteins related to cell proliferation and differentiation such as A0A480UJS2 and A0A480Q1D2 were up-regulated in uteri of the ZEA 3.0 group, while tumor suppressor-related proteins A0A286ZLG5 (NBL1), A0A4X1V1P1 (GPC6), A0A4X1T3Y9, and A0A481D9A4 were down-regulated. The proteins A0A287AL41 (PACRG) and A0A287BK52 (SUGP1) involved in gene expression regulation in the ZEA 3.0 group were up-regulated and down-regulated, respectively. In addition, the cell structure protein A0A480PQZ4 was up-regulated and A0A4X1TQZ8 (INA) was down-regulated in the ZEA 3.0 group compared with the control group. It is also worth noting that hormone secretion protein Q9GLG4 (CHGB), and apoptosis proteins such as A0A480UYE2, A0A5G2RN58 (ALDH1A2), and A5A8Y8 (EGFL8) were all down-regulated in the ZEA 3.0 group compared with the control group.

### 2.7. Gene Ontology (GO) Function Annotation of DEPs

**Figure 3:**
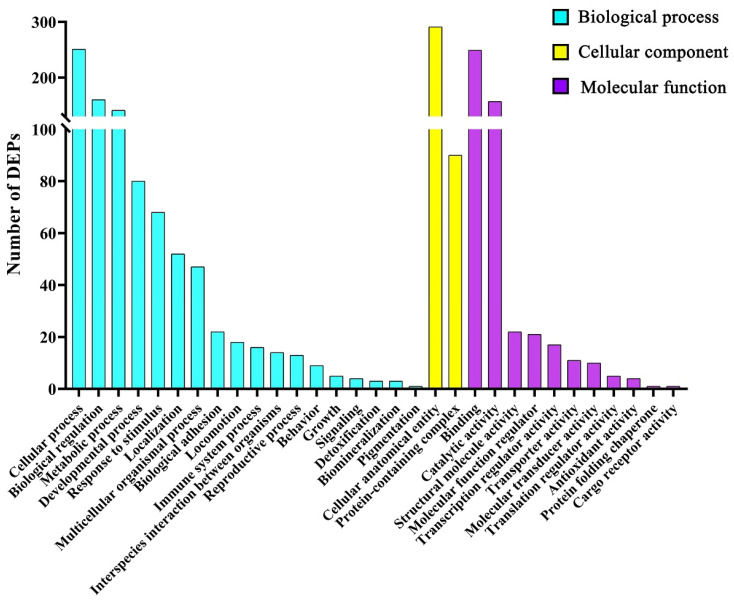
The GO function annotation analysis of differentially expressed proteins of uteri in gilts from the control and ZEA3.0 treatment group. The abscissa represent the GO secondary classification.

GO function annotation was performed based on biological process (BP), cellular component (CC), and molecular function (MF) with DEPs in the control and ZEA 3.0 treatment groups (Figure 3, Table S4). In the BP category, the number of DEPs enriched in the cellular process, biological regulation, and metabolic process was more than that of the other 15 categories. The DEPs are only enriched in the cellular anatomical entity and protein-containing complex in the CC category, and DEPs were mainly enriched in binding and catalytic activity terms in the MF category.

### 2.8. Kyoto Encyclopedia of Genes and Genomes (KEGG) Enrichment Analysis of DEPs

**Figure 4:**
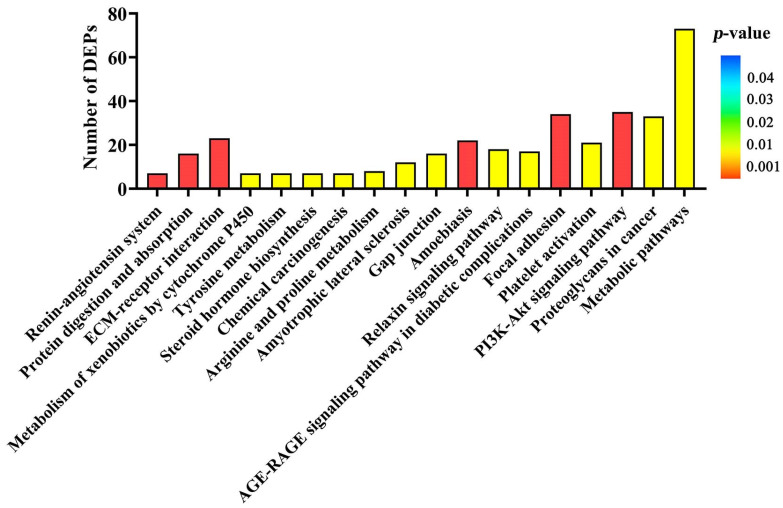
The KEGG enrichment analysis of differentially expressed proteins (DEPs) of uteri in gilts from the control and ZEA3.0 treatment groups. The abscissa from left to right indicate the enrichment rate of the KEGG pathway from high to low.

A total of 42 functional pathways were significantly enriched with DEPs in KEGG pathway analysis (*p* < 0.05, Table S5). Figure 4 showed the top 18 functional pathways with significant enrichment of DEPs (*p* < 0.01), which included ECM-receptor interaction, protein digestion and absorption, focal adhesion, the PI3K-AKT signaling pathway, renin-angiotensin system, amoebiasis, gap junction, proteoglycans in cancer, platelet activation, the relaxin signaling pathway, amyotrophic lateral sclerosis, the AGE-RAGE signaling pathway in diabetic complications, metabolism of xenobiotics by cytochrome P450, arginine and proline metabolism, tyrosine metabolism, steroid hormone biosynthesis, metabolic pathways, and chemical carcinogenesis, in order of significance from high to low.

### 2.9. Verification of DEPs

**Figure 5:**
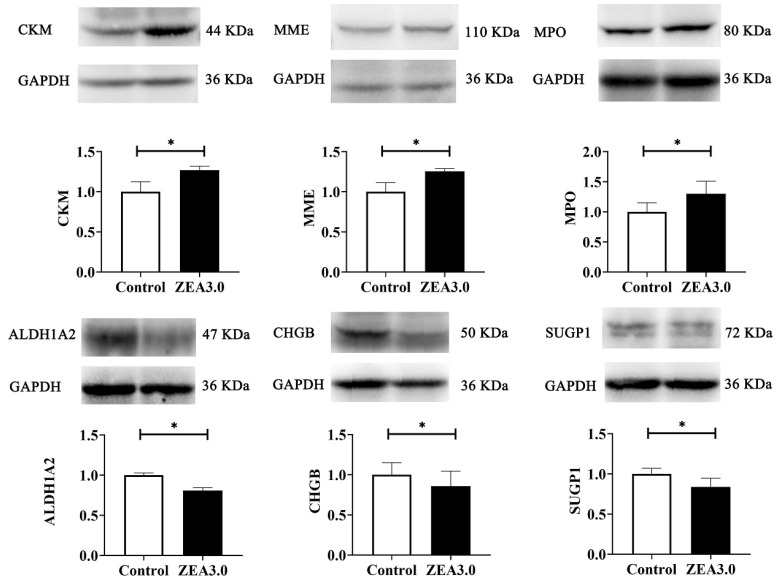
Western blot analysis of uterus proteins in gilts from the control and ZEA3.0 treatment groups (*n* = 3). Glyceraldehyde-3-phosphate dehydrogenase (GAPDH) was selected as the internal reference protein for western blot. CKM, creatine kinase M-type; MME, atriopeptidase; MPO, myeloperoxidase; ALDH1A2, aldehyde dehydrogenase 1 family member; CHGB, secretogranin-1; SUGP1, SURP and G-patch domain containing 1. * Means differ significantly (*p* < 0.05).

To validate the proteomics results, western blot was performed to evaluate the expression levels of six selected DEPs, and glyceraldehyde-3-phosphate dehydrogenase (GAPDH) was selected as the internal reference protein (Figure 5). Compared with the control group, the protein expressions of creatine kinase M-type (CKM), atriopeptidase (MME), and myeloperoxidase (MPO) in the uteri of gilts fed with 3.0 mg/kg of ZEA diet were increased (*p* < 0.05), whereas the expression levels of aldehyde dehydrogenase 1 family member (ALDH1A2), secretogranin-1 (CHGB), and SURP and G-patch domain containing 1 (SUGP1) were decreased (*p* < 0.05). The results of the six selected proteins are consistent with proteomics (Table 6 and Table S2).

## 3. Discussion

The effect of ZEA-contaminated diet on growth performance of pigs was inconsistent. Previous researchers reported that ZEA at doses of 5, 10, and 15 μg/kg BW increased the BW of 14.5 kg gilts [21], and Jiang et al. [22] reported that 1 mg/kg of ZEA-contaminated diet increased the ADG and ADFI of weaned gilts. The structure of ZEA is analogous to 17β-estradiol, and ZEA can bind to estrogen receptors to play the role of estrogen [2,23]. Moreover, estrogen plays an important role in body growth and skeletal maturation [24,25], and it could enhance the growth velocity of the pubertal body [26]. Therefore, weight gain caused by ZEA may be related to the effect of estrogen. To the contrary, it was reported that pigs fed with 0.2 to 0.8 mg/kg of ZEA had reduced ADG and gain/feed (G/F), whereas they had increased ADFI [27]. Weaver et al. [28] showed increased ADFI and decreased ADG of pigs were caused by deoxynivalenol (DON) (4.8 mg/kg) and ZEA (0.3 mg/kg) co-contaminated diets. Both ZEA and DON decreased the growth performance of pigs, which might be due to their interference with immune regulation, destruction of intestinal barrier function and cell death [29]. The reasons of ADFI increase were not clear. Wang et al. [27] suggested that feed palatability was not decreased with ZEA and the increase of ADFI might compensate for reduction of digestible nutrients after pigs were fed the ZEA-contaminated diet. In this study, no difference in ADFI, ADG and F/G of 3.0 mg/kg ZEA diet was observed, and the results were consistent with those of Jiang et al. [30] and Liu et al. [31]. In addition, ADG in the ZEA 3.0 group showed a statistical trend compared with the control group. The increase of ADG may be due to the synthetic effect of ZEA against toxicity. Meanwhile, the difference of growth performance of pigs might be caused by the doses of ZEA, age, BW and so on.

Vulva development is related to estrogen in puberty [32]. A study by Stob et al. [4] showed that ZEA could induce vulva swelling and reproductive tract prolapse. Oliver et al. [33] reported increased vulva size without increased BW gain and increased reproductive tract weight after pre-pubertal or pubertal gilts were fed 1.5 mg/g of ZEA-contaminated diet. The vulva is a part of external extension of the reproductive tract; the increased vulva size might be attributed to estrogen effect of ZEA promoting the reproductive tract growth of gilts. ZEA at 1.04 mg/kg could increase vulva size and other vulva indexes [34]. Vulva length, width and area were linearly increased after gilts fed ZEA diet at levels of 0.2, 0.4 and 0.8 mg/kg [27]. Wu et al. [35] observed increased vulva size of gilts fed 0.8 and 1.6 mg/kg ZEA-contaminated diet. Jiang et al. [30] reported that the vulva size of 2 and 3.2 mg/kg ZEA groups was increased compared with 0 mg/kg ZEA; this level of ZEA (3.2 mg/kg) was similar to our dose. In our study, the final vulva area, final area/initial area and final area/final weight of gilts in the ZEA 3.0 group were greater than those in the control group, which indicates that ZEA at 3.0 mg/kg showed a significant estrogen effect. Therefore, the vulva size can be used as a clinical indicator of gilts fed ZEA-contaminated diet.

The uterus was considered to be the target organ for ZEA toxicity; it may be more susceptible to ZEA [1]. Chen et al. [10] showed that the uterine size of gilts increased linearly with the increase of ZEA doses (0, 1.1, 2.0 and 3.2 mg/kg). Similarly, we found that uterine indexes of gilts (UL, UW, UW/UL and UW/final weight) in the ZEA3.0 group were higher than those in the control group. In addition, Zhou et al. [11] reported that the thickness of lamina propria and myometrium and the number of uterus glands were increased after gilts were fed the 1.0 mg/kg ZEA-contaminated diet or received intramuscular injection estradiol at 0.75 mL (1.5 mg); these results were consistent with our studies. Meanwhile, Song et al. [13] reported that ZEA could change GSK-3β and Cyclin D1 relative mRNA and protein expressions via the Wnt/β-catenin signaling pathway in porcine endometrial epithelial cells. Zhou et al. [18] found that the TGF-β1/Smad3 signaling pathway was activated and PCNA, BAX and BCL-2 relative mRNA and protein expressions were increased in uteri after gilts were fed ZEA-contaminated diet. These results indicated that ZEA might play the role of estrogen to activate certain signaling pathways, thereby inducing swelling the uterus in gilts. However, the molecular mechanism of ZEA-induced uterine development remains unclear and needs further study.

Current studies have shown that ZEA and its metabolites are external steroid hormones that could interfere with serum hormones levels [2]. He et al. [36] reported that the content of follicle stimulating hormone was significantly reduced after ovariectomized pigs were injected with ZEA at doses of 7.5 mg/kg BW. Compared with the control group, 2 mg/kg of ZEA could decrease the content of luteinizing hormone in pre-pubertal gilts [37]. Moreover, studies by Su et al. [38] showed that 1 mg/kg of ZEA could reduce the contents of follicle-stimulating hormone and luteinizing hormone in serum of gilts. These results were consistent ours. Yang et al. [3] reported that ZEA could play an estrogenic role in inducing the reproductive organs development and central precocious via hypothalamic kisspeptin-GPR54 signaling, and reduced the content of gonadotropin-releasing hormone in immature female rats. ZEA and α-ZOL could decrease the content of follicle stimulating hormone via the GPR30 receptor of the pituitary in gilts [36]. These results indicated that ZEA might play an estrogenic effect in the hypothalamo-pituitary-gonadal axis. In our study, the decreased contents of follicle-stimulating hormone, luteinizing hormone and gonadotropin-releasing hormone might be due to the feedback effect of hypothalamo-pituitary-gonadal axis. The ZEA (0.2 mg/kg) could reduce the contents of gonadotropin and gonadotropin-releasing hormone [39]. Previous study reported that 2 mg/kg of ZEA could decrease estradiol in gilts compared with control gilts [37]. Jiang et al. [40] and Su et al. [38] reported that the contents of estradiol and progesterone were reduced after gilts were fed 1 mg/kg of ZEA contaminated diet. Productions of estradiol and progesterone were mainly carried out with ovaries in puberty [41]. With the increase of ZEA (0.5, 1 and 1.5 mg/kg), the ovarian size increased first and then decreased [42]. Wan et al. [43] observed the increased quantity of atretic growing follicles, but a greater volume and quantity of healthy growing follicles in the deep ovarian cortex with an increasing level of dietary ZEA (0, 0.15, 1.5 and 3.0 mg/kg). The absence of a difference in estradiol and progesterone in this study might be attributed to that the estradiol and progesterone secreted by healthy growing follicles in the deep ovarian cortex could offset the insufficient secretion of atretic growing follicles. Therefore, the effect of ZEA on serum hormone levels needs further study.

Previous studies reported that the α-ZOL and β-ZOL were the main metabolites of ZEA in pigs [44,45]. Studies showed that ZEA, α-ZOL, and β-ZOL could be metabolized by enterohepatic cycling and biliary excretion in the body, thereby entering the blood, tissues and organs [46,47]. ZEA, α-ZOL, and β-ZOL were detected in serum after pre-pubertal gilts were fed ZEA at doses of 5, 10 and 15 μg /kg BW for 21 and 42 days [48]. The contents of ZEA, α-ZOL, and β-ZOL in serum of ZEA treatment pigs (doses at 0.15, 1.5 and 3 mg/kg) were higher than those in control pigs [43]. Zöllner et al. [44] reported that ZEA and its metabolites (α-ZOL and β-ZOL) were detected in urine, muscle tissue and liver of pigs after feeding them mycotoxin-contaminated oats. ZEA and α-ZOL in the reproductive tract, uterus, ovary and liver of swine fed 6 mg/kg of ZEA diet could be detected [49]. In our study, ZEA, α-ZOL, and β-ZOL in uteri of gilts fed 3.0 mg/kg ZEA were higher those in control gilts, and the results confirmed that the uterus was the target organ of ZEA.

There are very complex reasons that ZEA induces development of the uterus in pre-pubertal or pubertal gilts when the expressions of diverse microRNAs, mRNAs, and proteins change [12,13,50]. Revealing the mechanism underlines the changes in uterine development induced by ZEA is crucial for elucidating the molecular mechanism of ZEA and therefore eliminating the adverse effects of ZEA. In the past decade, more abnormal physiological mechanisms and animal metabolism were revealed via MS-based proteomic techniques [15,17], although research on ZEA-induced uterine development were rare. Compared with the control group, 423 DEPs were identified by TMT-labeled quantitative proteomics in the ZEA 3.0 group, of which 291 and 132 DEPs were up-regulated and down-regulated, respectively. The confirmed up-regulated expression of CKM, MME, and MPO and down-regulated expression of ALDH1A2, CHGB, and SUGP1 by western blot indicated that our proteomics data were dependable. CKM was a catalytic activity protein that can reversibly catalyze various phosphogens between ATP and ADP, thereby playing an important role in maintaining the cellular energy homeostasis [51]. Moreover, Xiong et al. [52] reported that CKM was highly expressed in skeletal muscle and myocardium, and its gene expression was increased after muscle cell differentiation. Wan et al. [43] reported that ZEA could promote follicle development via the SIRT1/PGC-1α signaling pathway related to energy metabolism. MME was a membrane-bound metallopeptidase whose main functions involved immune response and cell proliferation [53]. MME could catalyze the activity of the PENT tumor suppressor protein and could inhibit the phosphoinositide 3-kinase/protein kinase B (PI3K/AKT) signaling pathway to reduce cell survival and migration in cancer growth [54]. More studies reported that the PI3K/AKT signaling pathway was activated in cancer cells or tumors, because the phosphorylated PI3K and AKT proteins could promote cell survival, cycle, and proliferation [55]. MPO is a member of the heme peroxidase family and is mainly related to oxidation capacity and immunity [56]. A previous study reported that MPO could catalyze H_2_O_2_ and chloride to produce HOCl, thereby inducing cell damage, oxidative stress, and inflammation [57]. ZEA (7 μg/mL and 8 μg/mL) can promote ROS production and induce oxidative stress by damaging antioxidant enzyme activity in porcine IPEC-J2 cells [6]. ALDH1A2, a member of the aldehyde dehydrogenase (ALDH) superfamily, catalyzes the synthesis of retinoic acid (RA), which played a role in cell differentiation, growth and adaptive immune responses [58]. A study showed that up-regulated ALDH1A2 suppressed ovarian cancer cell proliferation via activation of transcription 3 (STAT3) [59]. Choi et al. [60] reported that down-regulated ALDH1A2 enhanced the proliferation of ovarian cancer cells. Pajewska et al. [61] observed ZEA and its metabolites in endometrial cancer tissues. High levels of estrogen induces endometrial carcinoma (type I) [62]. Therefore, ALDH1A2 might function as a potential tumor suppressor protein in the process of ZEA 3.0 treatment. CHGB participates in the biogenesis, maturation and release of secretory granules in endocrine cells [63]. Uterine glands can synthesize and secrete a variety of enzymes, growth factors, hormones and other substances [64]. SUGP1 is an essential gene for coding splicing proteins [65]. Alsafadi et al. [66] reported that SUGP1 loss and mutations induced abnormal splicing of SF3B1 (core subunit of spliceosome component) in lung adenocarcinoma. In the present study, increased CKM, MME, and MPO, and decreased ALDH1A2, CHGB, and SUGP1 might be related to energy metabolism, oxidative stress, proliferation or apoptosis, immune reaction and various adverse effects induced by ZEA. However, these changes in DEP expression need further study.

Cell cycle dysregulation is a hallmark of cell proliferation or apoptosis, and CDK7 can regulate the transcription cycle of RNA polymerase II and promote the cell cycle via activation of the T-loop [67]. The CDK7 is highly expressed in non-small cell lung cancer, silencing and inhibiting CDK7-inhibited tumor growth [68]. CDK7 was significantly up-regulated in gilts after ZEA 3.0 treatment in the present study, and the uterine development in gilts after ZEA treatment might be due to the heightened velocity of the cell cycle induced by ZEA. In our current study, the DEPs were mainly clustered into the biological process categories including cellular process, biological regulation, and metabolic process. It was worth noting that estrogen has the functions of promoting the metabolic process, accelerating the cell cycle and regulating cell proliferation [69]. The enhanced metabolic process and the accelerated cell cycle might be caused by changes in protein expression related to cell proliferation, differentiation, and growth function induced by ZEA, which suggests that uterine cells such as lamina propria cells, myometrium cells, and endometrial epithelial cells of gilts treated by ZEA might be more likely to survive or have stronger biological activity, including cell cycle, proliferation, differentiation, and growth function, than normal uterine cells.

Another aim of our current study was to identify the important signaling pathways involved in DEPs induced by ZEA during uterine development. Interestingly, several metabolic signaling pathways such as metabolism of xenobiotics by cytochrome P450, tyrosine metabolism, metabolic pathways, and arginine and proline metabolism were found. The ECM-receptor interaction, focal adhesion, and PI3K-AKT signaling pathway were selected to account for uterine development in gilts treated with ZEA3.0. The extracellular matrix (ECM) is a highly dynamic structural network, which includes many elements such as collagens, fibronectin laminins and growth factors [70]. Cell invasiveness and proliferation of hepatocellular carcinoma and prostate cancer result from the activation of ECM-receptor [70,71]. We found that several collagen domain-containing DEPs were clustered in ECM-receptor interaction and focal adhesion, such as COL7A1, COL5A3, COL5A2, COL3A1, and COL1A2. When focal adhesion is connected through the ECM with the cytoskeletons of cells, it senses the changes signals in the ECM and bidirectionally regulates the cell and ECM [72]. A study reported that metastatic inhibition of colorectal cancer cells was carried out via regulation of ECM-receptor interaction and focal adhesion [73]. Therefore, activation of ECM-receptor interaction and focal adhesion might be one of the reasons for ZEA-induced uterine development in pre-pubertal gilts in this study. Moreover, many studies have reported that estrogen or estrogen receptors could activate PI3K-AKT signaling pathway and induce cell survival and proliferation. Proliferation of breast cancer cells was achieved through activation of PI3K-AKT-NF-κB signaling under the estrogen effect [74]. Similarly, activation of G protein-coupled estrogen receptor 1 (GPER1) suppressed autophagy of cardiomyocyte via PI3K-AKT-mTOR signaling [75]. For further study, in vitro experiments will reveal whether ZEA induces uterine development through the PI3K-AKT signaling pathway.

## 4. Conclusions

Our results strongly suggested that ZEA could be transformed to α-ZOL and β-ZOL. ZEA and its metabolites could be accumulated in uteri of gilts after being fed ZEA-contaminated feed orally. Reduced serum follicle stimulation hormone, luteinizing hormone and gonadotropin-releasing hormone concentrations in gilts with ZEA 3.0 treatment were observed. Meanwhile, ZEA might change gene expression in the cellular process, biological regulation and metabolic process by interfering with serum hormones or through the direct effects of ZEA and its metabolites on the uterine target organ, thus promoting the development of the vulva and uterus. However, further studies are needed to elucidate these mechanisms.

## 5. Materials and Methods

### 5.1. Animals, Treatments, Management

A total of 16 healthy weaned gilts (Duroc × Landrace × Yorkshire) at 35 d with an average BW of 12.45 ± 0.19 kg (mean ± SEM) were randomly divided into two groups. Gilts were individually housed in stainless-steel cages (0.48 m^2^) with a nipple drinker, feed trough and plastic-slatted floors. All animals had free access to water and feed, and the experiment was performed at the Animal Research Station of Shandong Agricultural University (Tai’an, China). The control feed was the basal diet formulated according to the NRC (2012, Table 7) [76], and the ZEA 3.0 feed was the basal diet supplemented with 3.0 mg/kg of ZEA with a purity of 98% purchased from Fermentek (Jerusalem, Israel). The dissolution of purified ZEA was performed with ethyl acetate, which sprayed on talcum powder for preparation of 1000 mg/kg ZEA premix. The 10 mg/kg ZEA corn premix was prepared by diluting ZEA premix (1000 mg/kg) with corn flour without toxins. The 3.0 mg/kg ZEA feed was obtained according to the instructions by Liu et al. [31]. The room and equipment was fully cleaned and disinfected before the start of this experiment, and disinfection for room and gilts was performed every week during the experimental period. The room temperature was controlled at approximately 30 °C with a 7-day adaptation period and then at 26–28 °C for the 32-d experimental period. In addition, the relative humidity was kept at about 65% during the experimental period.

### 5.2. Mycotoxins Determination

The content of mycotoxins of the experimental diet were determined by the Qingdao Entry-Exit Inspection and Quarantine Bureau, according to Zhou et al. [12] and Liu et al. [31]. The detection limit of ZEA, fumonisin, aflatoxin, and deoxynivalenol were 0.01 mg/kg, 1.0 µg/kg, 0.1 mg/kg, 1.0 µg/kg, and 0.05 mg/kg, respectively. Mycotoxins of six duplicate feed samples from each treatment were determined. The ZEA level of the ZEA 3.0 diet was 3.12 ± 0.13 mg/kg. ZEA and other mycotoxins in the control diet were not detected.

### 5.3. Growth Performance

On the first and final day of this experiment, the weight of gilts was measured and the ADG was calculated. The ADFI was recorded for each gilt. Then, F/G was calculated by ADFI/ADG.

### 5.4. Vulva Size Measurement

The vulva width and length of gilts were measured with Vernier calipers every 3 days during the experiment period, and vulva size was calculated according to the methods of Jiang et al. [30] and Wan et al. [34].

### 5.5. Sample Collection

Blood samples were collected into 10 mL sterile non-heparinized tubes from the jugular vein of gilts after fasting for 12 h at the end of the feeding experiment. Then, the blood samples were centrifuged at 3000× *g* for 15 min to separate the serum, which was transferred into 1.5 mL sterile Eppendorf tubes and stored in a −20 °C freezer for determination of estradiol, progesterone, follicle-stimulating hormone, luteinizing hormone and gonadotropin-releasing hormone.

The abdominal cavities of gilts were immediately opened after gilts were injected intramuscularly with 0.1 mg/kg BW Zoletile 50 Vet (Virbac, Carros, France), and the uterus was isolated. The UL and UW were measured, and the UL/UW and UW/final weight of each gilt were calculated. Three uterine sample of each gilt were collected, and one was fixed in 4% paraformaldehyde for 48 h to observe the uterine development; the other two were stored at −80 °C for proteomic analysis and western blot verification.

### 5.6. Serum Hormone Measurement

Serum estradiol, progesterone, follicle stimulating hormone, luteinizing hormone, and gonadotropin releasing hormone were determined using radioimmunoassay (RIA) [^125^I] kits (Nanjing Jiancheng Bioengineering Institute, Nanjing, China) according to the instructions by Wan et al. [34] and Jiang et al. [40].

### 5.7. Mycotoxins Detection in the Uterus

The 0.4 g frozen uterine sample in 5 mL sterile centrifuge tube was ground with a high-throughput tissue grinder (Scientz-192, Beyotime, Shanghai, China). The homogenate was centrifuged at 10,000× *g* at 4 °C for 10 min, and the supernatant was separated to detect ZEA, α-ZOL, and β-ZOL by LC-MS/MS at the Institute of Quality Standards and Detection Technology of the Chinese Academy of Agricultural Sciences, according to Wan et al. [43] and Zhang et al. [77]. The LC-MS/MS analysis was performed using Agilent 1200 liquid chromatograph (Agilent Technologies, Palo Alto, CA, USA) installed with the 3200 QTrap^®^ mass spectrometry system (Applied Biosystems, Foster City, CA, USA).

### 5.8. Uterine Development Index Measurement

The uterus tissue was dehydrated successively in ethanol (60%, 70%, 80%, 90%, 95%, and 100%) and xylene solutions for 1.5 h, after fixation in 4% paraformaldehyde for 48 h. Subsequently, it was embedded in the paraffin wax, and cut to a 4-μm section that was mounted on a poly-L-lysine-coated glass slide using Leica RM 2235 microtome (Leica, Wetzlar, Germany), according to the methods of Zhang et al. [78]. The sections of uterus were stained with hematoxylin and eosin, and sealed with neutral resin. Three uterine sections from each gilt were selected to measure the thickness of lamina propria and myometrium using a microscope and image analysis software (Olympus BX41, Tokyo, Japan). The number of uterus glands was recorded using image analysis software through visual observation.

### 5.9. Protein Sample Preparation (TMT Quantitative Proteomic)

The frozen uterine samples were ground with liquid nitrogen and transferred into radio immunoprecipitation assay (RIPA) lysis buffer (WB100, New Cell & Molecular Biotech, Shanghai, China) containing 1% sodium deoxycholate (SDS), 8 M urea and 2% protease and phosphatase inhibitor (P1048, Beyotime, Shanghai, China). The uterine samples were placed on ice for 30 min, sonicated for 3 min and then centrifuged at 16,000× *g* at 4 °C for 30 min. The uterine protein concentration was determined using a bicinchoninic acid (BCA) protein assay Kit (WB6501, New Cell & Molecular Biotech, Shanghai, China) and the protein was separated by SDS-polyacrylamide gel electrophoresis (SDS-PAGE).

### 5.10. Protein Digestion

Triethylammonium bicarbonate buffer (TEAB) was added to the uterine protein samples to reach the final concentration of 100 mM. Tris(2-carboxyethyl)phosphine (TCEP) was added to each protein sample for final concentration of 10 nM, and the protein samples were incubated at 37 °C for 1 h. The iodoacetamide (IAM) was added to the uterine protein samples with a final concentration of 40 mM, and the uterine protein samples were incubated at 25 °C for 40 min shielded from light. Protein samples were diluted with the acetone at 6-fold volume and were placed in −20 °C for 4 h. The protein samples were centrifugated at 10,000× *g* at 4 °C for 20 min, and were dissolved by the 100 μL of 100 mM TEAB. Finally, they were digested overnight with trypsin at 37 °C to produce peptides.

### 5.11. TMT-Labeled Peptides

The peptides were labeled using a TMT 10Plex^TM^ kit (90111, Thermo Fisher Scientific, Shanghai, China), according to the manufacturer’s protocol. UC11, UC21, UC31, and UC41 of the control group were labeled as TMT10-127N, TMT10-127C, TMT10-128N, and TMT10-128C, and UT11, UT21, UCT1, and UT41 of the ZEA 3.0 group were labeled as TMT10-129N, TMT10-129C, TMT10-130N, and TMT10-130C, respectively. All the labeled samples were mixed in equal quantities and concentrated in vacuum.

### 5.12. LC-MS/MS Analysis

After each sample was dissolved with 2% acetonitrile, the ACQUITY UPLC BEH C18 column (1.7 μm, 2.1 mm × 150 mm, Waters, Milford, MA, USA) was used for fractionation. LC-MS analysis of the labeled peptides was performed on a Q-Exactive HF-X mass spectrometer coupled to an EASY-nLC 1200 (Thermo Fisher Scientific, San Jose, CA, USA). The labeled peptides were loaded onto the C18-reversed-phase column (25 cm × 75 µm, Thermo Fisher Scientific, San Jose, CA, USA), chromatographed in buffer A (2% acetonitrile and 0.1% formic acid), and separated with a linear gradient of buffer B (80% acetonitrile and 0.1% formic acid) for 120 min at a flow rate of 300 nL/min. The linear elution gradient was set as follows: 0 to 65 min, 5% to 23% buffer B; 65 to 79 min, 23% to 29% buffer B; 79 to 88 min, 29% to 38% buffer B; 88 to 90 min, 38% to 48% buffer B; 90 to 92 min, 48% to 100 buffer B; buffer B was maintained at 100% for 92 to 120 min. The MS acquisition was carried out in DDA (data dependent acquisition) mode and the data-dependent top-20 acquisition mode. The MS/MS spectrum was obtained by automatic switching between MS and MS/MS. A full MS scan ranging from 350 to 1500 *m*/*z* was conducted at a resolution of 120,000 with an automatic gain control (AGC) target value of 3 × 10^6^ ions and a maximum ion transfer (IT) of 20 ms. The precursor ions were fragmented by means of high energy collisional dissociation (HCD), and all MS/MS spectrum were scanned using the following parameters: resolution 45,000; AGC 1 × 10^4^ ions; maximum IT 50 ms; dynamic exclusion duration 20 s; intensity threshold 8.3 × 10^4^.

### 5.13. Database Search

The protein database (uniprot-taxonomy-9823.unique.fasta) was selected for peptide and protein identification. The raw data of the LC-MS/MS were analyzed using ProteomeDiscoverer^TM^ Software 2.4 (Thermo Fisher Scientific, San Jose, CA, USA). Trypsin (Full) was designated as a cleavage enzyme allowing up to two missing cleavages, and the fixed modification was set to cysteine alkylation. The dynamic modification was set as follows: oxidation (M), acetyl (protein N-terminus), met-loss (protein N-terminus), met-loss + acetyl (protein N-terminus). The static modification was set to: carbamidomethyl (C), TMT 6plex (K), TMT 6plex (N-terminus). The mass tolerance for the precursor was adjusted to 20 mg/kg, and the mass tolerance for the fragment was set as 0.02 Da. In addition, the validation was based on q-value. For peptide and protein identification, the false discovery rate (FDR) was set to 1%. At least one unique peptide was analyzed for protein identification.

### 5.14. Bioinformatics Analysis

The data for the identified differentially expressed proteins (DEPs) of uterine samples were used for GO terms (http://www.geneontology.org/, accessed on 28 July 2021) function annotation analysis. The DEPs were assigned into three categories of ontology—BP, CC and MF—by the GO function annotation analysis. To reveal the potential functions of the DEPs, the data of the DEPs were submitted to KEGG (http://www.genome.jp/kegg/, accessed on 28 July 2021) for KEGG enrichment analysis.

### 5.15. Western Blot Verification

After the protein concentration of uterine samples were determined with BCA kits, all the protein samples were diluted to 55 µg with RIPA lysis buffer and separated by SDS polyacrylamide gel electrophoresis (SDS-PAGE). The isolated proteins were transferred to polyvinylidene fluoride (PVDF) membranes (ISEQ00010, Solarbio, Beijing, China). The PVDF membranes were incubated in the rapid blocking buffer (P30500, New Cell & Molecular Biotech, Shanghai, China) for 15 min, and washed three times with the tris-buffered saline containing Tween (WB21000, New Cell & Molecular Biotech, Shanghai, China). The primary antibodies and anti-rabbit IgG (1:2000, A0208, Beyotime, Shanghai, China) were diluted using the universal antibody diluent (WB500D, New Cell & Molecular Biotech, Shanghai, China). The PVDF membranes were incubated with the following primary antibodies: GAPDH (1:5000, AF1186, Beyotime, Shanghai, China), anti-CKM (1:1000, P06732, Cusabio, Wuhan, China), anti-MME (1:500, P08473, Cusabio, Wuhan, China), anti-MPO (1:500, P05164, Cusabio, Wuhan, China), anti-ALDH1A2 (1:500, O94788, Cusabio, Wuhan, China), anti-CHGB (1:500, P05060, Cusabio, Wuhan, China), and anti-SUGP1 (1:500, Q8IWZ8, Cusabio, Wuhan, China) at 4 °C overnight. The PVDF membranes were washed three times and incubated in anti-rabbit IgG at 4 °C for 2 h. The PVDF membranes were immersed in a high-sensitivity luminescence reagent (P10100, New Cell & Molecular Biotech, Shanghai, China), exposed to film using FusionCapt Advance FX7 (Beijing Oriental Science and Technology Development Co. Ltd., Beijing, China) and analyzed using Image software (Image Pro-Plus 6.0, Media Cybernetics, Silver Spring, MD, USA).

### 5.16. Data Analysis

An individual gilt was regarded as the unit for all variables. Data are presented as mean ± SEM, after normal distribution assessment using Shapiro–Wilk’s statistic (*W* > 0.05). Data were statistically analyzed by Student’s T test with SAS 9.4 (Version 9.2, SAS Institute Inc., Cary, NC, USA). Treatment differences were considered statistically significant at *p* < 0.05. The screening criteria for DEPs were fold change (FC) > 1.2 or <0.83-fold and *p* < 0.05 in the control and ZEA 3.0 treatment group. The KEGG enrichment analysis was performed using Fisher’s exact test and was regarded as statistically significant at *p* < 0.05.

## Figures and Tables

**Table 1:** Effects of zearalenone (ZEA) on growth performance of weaned gilts ^1^.

Items	Control	ZEA3.0	*p*-Value
Initial body weight, kg	12.54 ± 0.38	12.36 ± 0.54	0.805
Final body weight, kg	28.44 ± 0.88	29.77 ± 0.90	0.309
Average daily feed intake, g	779.69 ± 16.93	821.19 ± 18.04	0.116
Average daily gain, g	496.88 ± 15.91	543.75 ± 16.83	0.062
Feed/gain	1.57 ± 0.02	1.52 ± 0.02	0.160

^1^ Treatments were basal diet supplemented with ZEA at the levels of 0 and 3.0 mg/kg, with analyzed ZEA concentrations of 0 and 3.12 ± 0.13 mg/kg, respectively. Data are expressed as mean value ± standard error (*n* = 8).

**Table 2:** Effects of zearalenone (ZEA) on vulva size of weaned gilts ^1^.

Items	Control	ZEA3.0	*p*-Value
Initial vulva area, cm^2^	0.76 ± 0.03	0.76 ± 0.05	0.972
Final vulva area, cm^2^	3.00 ± 0.08	10.69 ± 0.23	<0.001
Final area/initial area	4.00 ± 0.17	14.51 ± 0.94	<0.001
Final area/final weight, cm^2^/kg	0.11 ± 0.01	0.36 ± 0.02	<0.001

^1^ Treatments were basal diet supplemented with ZEA at the levels of 0 and 3.0 mg/kg, with analyzed ZEA concentrations of 0 and 3.12 ± 0.13 mg/kg, respectively. Data are expressed as mean value ± standard error (*n* = 8).

**Table 3:** Effects of zearalenone (ZEA) on serum hormones of weaned gilts ^1^.

Items	Control	ZEA3.0	*p*-Value
Estradiol, pmol/L	136.20 ± 4.00	127.85 ± 4.29	0.186
Progesterone, pmol/L	2035.47 ± 95.49	1850.92 ± 95.54	0.202
Follicle-stimulating hormone, U/L	10.64 ± 0.57	7.77 ± 0.40	0.004
Luteinizing hormone, pg/mL	368.99 ± 11.67	304.14 ± 14.78	0.006
Gonadotropin-releasing hormone, ng/L	20.22 ± 0.38	18.12 ± 0.45	0.006

^1^ Treatments were basal diet supplemented with ZEA at the levels of 0 and 3.0 mg/kg, with analyzed ZEA concentrations of 0 and 3.12 ± 0.13 mg/kg, respectively. Data are expressed as mean value ± standard error (*n* = 8).

**Table 4:** Effects of zearalenone (ZEA) on mycotoxins in the uteri of weaned gilts ^1^.

Items	Control	ZEA3.0	*p*-Value
ZEA, ng/g	0.00 ± 0.00	0.44 ± 0.01	<0.001
α-ZOL, ng/g	0.00 ± 0.00	0.94 ± 0.03	<0.001
β-ZOL, ng/g	0.00 ± 0.00	0.96 ± 0.03	<0.001

^1^ Treatments were basal diet supplemented with ZEA at the level of 0 and 3.0 mg/kg, with analyzed ZEA concentrations of 0 and 3.12 ± 0.13 mg/kg, respectively. Data were expressed as mean value ± standard error (*n* = 8).

**Table 5:** The top 10 up-regulated and top 10 down-regulated DEPs in gilts of the control and ZEA3.0 treatment groups ^1^.

Accession	Description	FC	*p*-Value
Q5XLD3	Creatine kinase M-type (CKM)	4.50	<0.001
F1RLE9	RING-type E3 ubiquitin transferase (TRIM36)	2.76	<0.001
A0A480UJS2	Laminin subunit alpha-5 isoform X1	2.54	0.002
A0A4X1UF72	Mitochondria-eating protein	2.53	0.020
A0A287AL41	Parkin coregulated (PACRG)	2.43	0.019
A0A287BPD6	Atriopeptidase (MME)	2.09	0.004
A0A5G2R354	Stabilizer of axonemal microtubules 2 (SAXO2)	2.09	0.003
A0A480PQZ4	Epiplakin isoform X1-like (Fragment)	2.08	0.001
A0A480Q1D2	Beta-parvin isoform X3	2.08	0.004
A0A4X1THU1	Uncharacterized protein	2.04	0.018
A0A286ZLG5	NBL1, DAN family BMP antagonist (NBL1)	0.53	0.011
A0A4X1V1P1	Uncharacterized protein (GPC6)	0.49	0.018
Q9GLG4	Secretogranin-1 (CHGB)	0.48	0.012
A0A5G2RN58	Aldehyde dehydrogenase 1 family member A2 (ALDH1A2)	0.45	0.033
A0A4X1T3Y9	ANK_REP_REGION domain-containing protein	0.45	0.016
A0A481D9A4	Olfactomedin-like protein 1 isoform X1	0.43	0.049
A5A8Y8	Epidermal growth factor-like protein 8 (EGFL8)	0.41	0.034
A0A480UYE2	Aminopeptidase	0.41	0.026
A0A4X1TQZ8	IF rod domain-containing protein (INA)	0.39	0.048
A0A287BK52	SURP and G-patch domain containing 1 (SUGP1)	0.32	0.017

^1^ Treatments were basal diet supplemented with ZEA at the levels of 0 and 3.0 mg/kg, with analyzed ZEA concentrations of 0 and 3.12 ± 0.13 mg/kg, respectively. The proteomic dates of 20 DEPs are shown in Table S2.

**Table 6:** Proteomics data for selected DEPs of uterus proteomics in gilts from the control and ZEA3.0 treatment groups ^1^.

Items	Control	ZEA3.0	FC	*p*-Value
CKM	1.00 ± 0.21	4.50 ± 0.42	4.50	<0.001
MME	1.00 ± 0.19	2.09 ± 0.14	2.09	0.004
MPO	1.00 ± 0.11	1.98 ± 0.33	1.98	0.029
ALDH1A2	1.00 ± 0.14	0.46 ± 0.05	0.46	0.012
CHGB	1.00 ± 0.20	0.45 ± 0.02	0.45	0.033
SUGP1	1.00 ± 0.12	0.32 ± 0.17	0.32	0.017

^1^ Treatments were basal diet supplemented with ZEA at the levels of 0 and 3.0 mg/kg, with analyzed ZEA concentrations of 0 and 3.12 ± 0.13 mg/kg, respectively. Data are expressed as mean value ± standard error (*n* = 4). CKM, creatine kinase M-type; MME, atriopeptidase; MPO, myeloperoxidase; ALDH1A2, aldehyde dehydrogenase 1 family member; CHGB, secretogranin-1; SUGP1, SURP and G-patch domain containing 1. The proteomic date of six DEPs are shown in Table S2.

**Table 7:** Ingredients and nutrient contents of the basal diet (air-dry basis).

Ingredients	Content %	Nutrients	Analyzed Values %
Expanded corn	64.43	Metabolizable energy, MJ/kg	13.86
Whey powder, CP 3%	5.0	Crude protein	18.48
Fermented soybean meal	14.0	Calcium	0.74
Expanded soybean	8.5	Total phosphorus	0.62
Fish meal, CP 63.28%	4.0	STTD phosphorus	0.41
CaHPO_4_	1.15	ATTD phosphorus	0.38
Pulverized limestone	0.7	Lysine	1.38
NaCl	0.2	Methionine	0.40
L-Lysine HCl	0.76	Sulfur amino acid	0.66
DL-Methionine	0.08	Threonine	0.85
L-Threonine	0.16	Tryptophan	0.23
L-Tryptophan	0.02		
Premix ^1^	1.00		
Total	100.00		

^1^ Supplied per kilogram of diet: vitamin A, 3300 IU; vitamin D_3_, 330 IU; vitamin E, 24 IU; vitamin K_3_, 0.75 mg; vitamin B_1_, 1.50 mg; vitamin B_2_, 5.25 mg; vitamin B_6_, 2.25mg; vitamin B_12_, 0.026 mg; pantothenic acid, 15.00 mg; niacin, 22.50 mg; biotin, 0.075 mg; folic acid, 0.45 mg; Mn (MnSO_4_·H_2_O), 4.00 mg; Fe (FeSO_4_·H_2_O), 90 mg; Zn (ZnSO_4_·H_2_O), 90 mg; Cu (CuSO_4_·5H_2_O), 6.00 mg; I (KIO_3_), 0.14 mg; Se (Na_2_SeO_3_), 0.30 mg. STTD, standardized total tract digestible; ATTD, apparent total tract digestible.

## Data Availability

In the present study, the proteomics data have been deposited to the ProteomeXchange Consortium (http://proteomecentral.proteomexchange.org) via the iProX partner repository with the dataset identifier PXD036461.

## Supplementary Materials

The following supporting information can be downloaded at: https://www.mdpi.com/article/10.3390/toxins14100692/s1, Table S1: Identified proteins; Table S2: Differentially expressed proteins; Table S3: Hierarchical clustering heat map data; Table S4: Gene ontology function annotation data; Table S5: Kyoto Encyclopedia of Genes and Genomes enrichment data.

## References

[1] Rai A., Das M., Tripathi A. (2020). Occurrence and toxicity of a fusarium mycotoxin, zearalenone. Crit. Rev. Food Sci. Nutr..

[2] Zheng W., Feng N., Wang Y., Noll L., Xu S., Liu X., Lu N., Zou H., Gu J., Yuan Y. (2019). Effects of zearalenone and its derivatives on the synthesis and secretion of mammalian sex steroid hormones: A review. Food Chem. Toxicol..

[3] Yang R., Wang Y.-M., Zhang L., Zhao Z.-M., Zhao J., Peng S.-Q. (2016). Prepubertal exposure to an oestrogenic mycotoxin zearalenone induces central precocious puberty in immature female rats through the mechanism of premature activation of hypothalamic kisspeptin-GPR54 signaling. Mol. Cell. Endocrinol..

[4] Stob M., Baldwin R., Tuite J., Andrews F., Gillette K. (1962). Isolation of an anabolic, uterotrophic compound from corn infected with *Gibberella zeae*. Nature.

[5] Zinedine A., Soriano J.M., Molto J.C., Manes J. (2007). Review on the toxicity, occurrence, metabolism, detoxification, regulations and intake of zearalenone: An oestrogenic mycotoxin. Food Chem. Toxicol..

[6] Fan W., Shen T., Ding Q., Lv Y., Li L., Huang K., Yan L., Song S. (2017). Zearalenone induces ROS-mediated mitochondrial damage in porcine IPEC-J2 cells. J. Biochem. Mol. Toxicol..

[7] Geng H., Tan X., Zhao M., Ma Y., Li Y. (2022). Proteomic analysis of zearalenone toxicity on mouse thymic epithelial cells. J. Appl. Toxicol..

[8] Fink-Gremmels J., Malekinejad H. (2007). Clinical effects and biochemical mechanisms associated with exposure to the mycoestrogen zearalenone. Anim. Feed Sci. Technol..

[9] Gajęcka M., Rybarczyk L., Jakimiuk E., Zielonka Ł., Obremski K., Zwierzchowski W., Gajęcki M. (2012). The effect of experimental long-term exposure to low-dose zearalenone on uterine histology in sexually immature gilts. Exp. Toxicol. Pathol..

[10] Chen X., Yang C., Huang L., Niu Q., Jiang S., Chi F. (2015). Zearalenone altered the serum hormones, morphologic and apoptotic measurements of genital organs in post-weaning gilts. Asian Australas. J. Anim. Sci..

[11] Zhou M., Yang L., Chen Y., Sun T., Wang N., Chen X., Yang Z., Ge J., Jiang S. (2019). Comparative study of stress response, growth and development of uteri in post-weaning gilts challenged with zearalenone and estradiol benzoate. J. Anim. Physiol. Anim. Nutr..

[12] Zhou M., Yang L., Shao M., Wang Y., Yang W., Huang L., Zhou X., Jiang S., Yang Z. (2018). Effects of zearalenone exposure on the TGF-β1/Smad3 signaling pathway and the expression of proliferation or apoptosis related genes of post-weaning gilts. Toxins.

[13] Song T., Yang W., Huang L., Yang Z., Jiang S. (2021). Zearalenone exposure affects the Wnt/β-catenin signaling pathway and related genes of porcine endometrial epithelial cells in vitro. Anim. Biosci..

[14] Johnson P.F., McKnight S.L. (1989). Eukaryotic transcriptional regulatory proteins. Annu. Rev. Biochem..

[15] Tian J., Du J., Han J., Bao X., Song X., Lu Z. (2020). Proteomics reveals the preliminary physiological states of the spotted seal (*Phoca largha*) pups. Sci. Rep..

[16] Liu S., Zhang W., Zhang F., Roepstorff P., Yang F., Lu Z., Ding W. (2019). TMT-based quantitative proteomics analysis reveals airborne PM_2.5_-induced pulmonary fibrosis. Int. J. Environ. Res. Public Health.

[17] Ma C., Wang W., Wang Y., Sun Y., Kang L., Zhang Q., Jiang Y. (2020). TMT-labeled quantitative proteomic analyses on the longissimus dorsi to identify the proteins underlying intramuscular fat content in pigs. J. Proteom..

[18] Li Y., Zhang B., Huang K., He X., Luo Y., Liang R., Luo H., Shen X.L., Xu W. (2014). Mitochondrial proteomic analysis reveals the molecular mechanisms underlying reproductive toxicity of zearalenone in MLTC-1 cells. Toxicology.

[19] González-Alvarez M.E., McGuire B.C., Keating A.F. (2021). Obesity alters the ovarian proteomic response to zearalenone exposure. Biol. Reprod..

[20] Soler L., Stella A., Seva J., Pallarés F.J., Lahjouji T., Burlet-Schiltz O., Oswald I.P. (2020). Proteome changes induced by a short, non-cytotoxic exposure to the mycoestrogen zearalenone in the pig intestine. J. Proteom..

[21] Rykaczewska A., Gajęcka M., Dąbrowski M., Wiśniewska A., Szcześniewska J., Gajęcki M.T., Zielonka Ł. (2018). Growth performance, selected blood biochemical parameters and body weights of pre-pubertal gilts fed diets supplemented with different doses of zearalenone (ZEN). Toxicon.

[22] Jiang S., Yang Z., Yang W., Yao B., Zhao H., Liu F., Chen C., Chi F. (2009). Effects of feeding purified zearalenone contaminated diets with or without clay enterosorbent on growth, nutrient availability, and genital organs in post-weaning female pigs. Asian Australas. J. Anim. Sci..

[23] Mahato D.K., Devi S., Pandhi S., Sharma B., Maurya K.K., Mishra S., Dhawan K., Selvakumar R., Kamle M., Mishra A.K. (2021). Occurrence, impact on agriculture, human health, and management strategies of zearalenone in food and feed: A review. Toxins.

[24] Lee P.A., Witchel S.F. (1997). The influence of estrogen on growth. Curr. Opin. Pediatr..

[25] Grumbach M.M. (2000). Estrogen, bone, growth and sex: A sea change in conventional wisdom. J. Pediatr. Endocr. Metab..

[26] Albin A.-K., Niklasson A., Westgren U., Norjavaara E. (2012). Estradiol and pubertal growth in girls. Horm. Res. Paediatr..

[27] Wang J., Chi F., Kim I. (2012). Effects of montmorillonite clay on growth performance, nutrient digestibility, vulva size, faecal microflora, and oxidative stress in weaning gilts challenged with zearalenone. Anim. Feed Sci. Technol..

[28] Weaver A.C., See M.T., Kim S.W. (2014). Protective effect of two yeast based feed additives on pigs chronically exposed to deoxynivalenol and zearalenone. Toxins.

[29] Holanda D.M., Kim S.W. (2021). Mycotoxin occurrence, toxicity, and detoxifying agents in pig production with an emphasis on deoxynivalenol. Toxins.

[30] Jiang S., Yang Z., Yang W., Gao J., Liu F., Broomhead J., Chi F. (2011). Effects of purified zearalenone on growth performance, organ size, serum metabolites, and oxidative stress in postweaning gilts. J. Anim. Sci..

[31] Liu X., Xu C., Yang Z., Yang W., Huang L., Wang S., Liu F., Liu M., Wang Y., Jiang S. (2020). Effects of dietary zearalenone exposure on the growth performance, small intestine disaccharidase, and antioxidant activities of weaned gilts. Animals.

[32] Farage M., Maibach H. (2006). Lifetime changes in the vulva and vagina. Arch. Gynecol. Obstet..

[33] Oliver W., Miles J., Diaz D., Dibner J., Rottinghaus G., Harrell R. (2012). Zearalenone enhances reproductive tract development, but does not alter skeletal muscle signaling in prepubertal gilts. Anim. Feed Sci. Technol..

[34] Wan B., Yuan X., Yang W., Jiao N., Li Y., Liu F., Liu M., Yang Z., Huang L., Jiang S. (2021). The effects of zearalenone on the localization and expression of reproductive hormones in the ovaries of weaned gilts. Toxins.

[35] Wu F., Cui J., Yang X., Liu S., Han S., Chen B. (2021). Effects of zearalenone on genital organ development, serum immunoglobulin, antioxidant capacity, sex hormones and liver function of prepubertal gilts. Toxicon.

[36] He J., Wei C., Li Y., Liu Y., Wang Y., Pan J., Liu J., Wu Y., Cui S. (2018). Zearalenone and alpha-zearalenol inhibit the synthesis and secretion of pig follicle stimulating hormone via the non-classical estrogen membrane receptor GPR30. Mol. Cell. Endocrinol..

[37] Wang D., Zhang N., Peng Y., Qi D. (2010). Interaction of zearalenone and soybean isoflavone on the development of reproductive organs, reproductive hormones and estrogen receptor expression in prepubertal gilts. Anim. Reprod. Sci..

[38] Su Y., Sun Y., Ju D., Chang S., Shi B., Shan A. (2018). The detoxification effect of vitamin C on zearalenone toxicity in piglets. Ecotoxicol. Environ. Saf..

[39] Parandin R., Behnam-Rassouli M., Mahdavi-Shahri N. (2017). Effects of neonatal exposure to zearalenone on puberty timing, hypothalamic nuclei of AVPV and ARC, and reproductive functions in female mice. Reprod. Sci..

[40] Jiang S., Yang Z., Yang W., Wang S., Liu F., Johnston L., Chi F., Wang Y. (2012). Effect of purified zearalenone with or without modified montmorillonite on nutrient availability, genital organs and serum hormones in post-weaning piglets. Livest. Sci..

[41] Taraborrelli S. (2015). Physiology, production and action of progesterone. Acta Obstet. Gynecol. Scand..

[42] Song T., Liu X., Yuan X., Yang W., Liu F., Hou Y., Huang L., Jiang S. (2021). Dose-effect of zearalenone on the localization and expression of growth hormone, growth hormone receptor, and heat shock protein 70 in the ovaries of post-weaning gilts. Front. Vet. Sci..

[43] Wan B., Huang L., Jing C., Li Y., Jiao N., Liang M., Jiang S., Yang W. (2022). Zearalenone promotes follicle development through activating the SIRT1/PGC-1α signaling pathway in the ovaries of weaned gilts. J. Anim. Sci..

[44] Zöllner P., Jodlbauer J., Kleinova M., Kahlbacher H., Kuhn T., Hochsteiner W., Lindner W. (2002). Concentration levels of zearalenone and its metabolites in urine, muscle tissue, and liver samples of pigs fed with mycotoxin-contaminated oats. J. Agric. Food Chem..

[45] Malekinejad H., Maas-Bakker R., Fink-Gremmels J. (2006). Species differences in the hepatic biotransformation of zearalenone. Vet. J..

[46] Biehl M.L., Prelusky D.B., Koritz G.D., Hartin K.E., Buck W.B., Trenholm H.L. (1993). Biliary excretion and enterohepatic cycling of zearalenone in immature pigs. Toxicol. Appl. Pharmacol..

[47] Shin B.S., Hong S.H., Bulitta J.B., Lee J.B., Hwang S.W., Kim H.J., Yang S.D., Yoon H.-S., Kim D.J., Lee B.M. (2009). Physiologically based pharmacokinetics of zearalenone. J. Toxicol. Environ. Health A.

[48] Rykaczewska A., Gajęcka M., Onyszek E., Cieplińska K., Dąbrowski M., Lisieska-Żołnierczyk S., Bulińska M., Babuchowski A., Gajęcki M.T., Zielonka Ł. (2019). Imbalance in the blood concentrations of selected steroids in pre-pubertal gilts depending on the time of exposure to low doses of zearalenone. Toxins.

[49] Pack E., Stewart J., Rhoads M., Knight J., De Vita R., Clark-Deener S., Schmale D.G. (2020). Quantification of zearalenone and alpha-zearalenol in swine liver and reproductive tissues using GC-MS. Toxicon X.

[50] Grenier B., Hackl M., Skalicky S., Thamhesl M., Moll W.-D., Berrios R., Schatzmayr G., Nagl V. (2019). MicroRNAs in porcine uterus and serum are affected by zearalenone and represent a new target for mycotoxin biomarker discovery. Sci. Rep..

[51] Ginevičienė V., Jakaitienė A., Utkus A., Hall E.C., Semenova E.A., Andryushchenko L.B., Larin A.K., Moreland E., Generozov E.V., Ahmetov I.I. (2021). CKM Gene rs8111989 Polymorphism and Power Athlete Status. Genes.

[52] Xiong Y., Zhuang R., Zhao G., Liu Y., Su Y., Wang W., Xi X., Yang Y., Han X., Xie S. (2022). Identification of the CKM Gene as a Potential Muscle-Specific Safe Harbor Locus in Pig Genome. Genes.

[53] Nalivaeva N., Zhuravin I., Turner A. (2020). Neprilysin expression and functions in development, ageing and disease. Mech. Ageing Dev..

[54] Sankhe R., Pai S.R.K., Kishore A. (2021). Tumour suppression through modulation of neprilysin signaling: A comprehensive review. Eur. J. Pharmacol..

[55] Lim W., An Y., Yang C., Bazer F.W., Song G. (2018). Trichlorfon inhibits proliferation and promotes apoptosis of porcine trophectoderm and uterine luminal epithelial cells. Environ. Pollut..

[56] Hawkins C.L., Davies M.J. (2021). Role of myeloperoxidase and oxidant formation in the extracellular environment in inflammation-induced tissue damage. Free Radic. Biol. Med..

[57] Chen S., Chen H., Du Q., Shen J. (2020). Targeting myeloperoxidase (MPO) mediated oxidative stress and inflammation for reducing brain ischemia injury: Potential application of natural compounds. Front. Physiol..

[58] Sanders S., Herpai D.M., Rodriguez A., Huang Y., Chou J., Hsu F.-C., Seals D., Mott R., Miller L.D., Debinski W. (2021). The Presence and Potential Role of ALDH1A2 in the Glioblastoma Microenvironment. Cells.

[59] Wang Y., Shao F., Chen L. (2018). ALDH1A2 suppresses epithelial ovarian cancer cell proliferation and migration by downregulating STAT3. OncoTargets Ther..

[60] Choi J.-A., Kwon H., Cho H., Chung J.-Y., Hewitt S.M., Kim J.-H. (2019). ALDH1A2 is a candidate tumor suppressor gene in ovarian cancer. Cancers.

[61] Pajewska M., Łojko M., Cendrowski K., Sawicki W., Kowalkowski T., Buszewski B., Gadzała-Kopciuch R. (2018). The determination of zearalenone and its major metabolites in endometrial cancer tissues. Anal. Bioanal. Chem..

[62] Murali R., Soslow R.A., Weigelt B. (2014). Classification of endometrial carcinoma: More than two types. Lancet Oncol..

[63] Yadav G.P., Zheng H., Yang Q., Douma L.G., Bloom L.B., Jiang Q.-X. (2018). Secretory granule protein chromogranin B (CHGB) forms an anion channel in membranes. Life Sci. Alliance.

[64] Gray C.A., Bartol F.F., Tarleton B.J., Wiley A.A., Johnson G.A., Bazer F.W., Spencer T.E. (2001). Developmental biology of uterine glands. Biol. Reprod..

[65] Sampson N.D., Hewitt J.E. (2003). SF4 and SFRS14, two related putative splicing factors on human chromosome 19p13. 11. Gene.

[66] Alsafadi S., Dayot S., Tarin M., Houy A., Bellanger D., Cornella M., Wassef M., Waterfall J.J., Lehnert E., Roman-Roman S. (2021). Genetic alterations of SUGP1 mimic mutant-SF3B1 splice pattern in lung adenocarcinoma and other cancers. Oncogene.

[67] Fisher R.P. (2019). Cdk7: A kinase at the core of transcription and in the crosshairs of cancer drug discovery. Transcription.

[68] Wang J., Zhang R., Lin Z., Zhang S., Chen Y., Tang J., Hong J., Zhou X., Zong Y., Xu Y. (2020). CDK7 inhibitor THZ1 enhances antiPD-1 therapy efficacy via the p38α/MYC/PD-L1 signaling in non-small cell lung cancer. J. Hematol. Oncol..

[69] Trenti A., Tedesco S., Boscaro C., Trevisi L., Bolego C., Cignarella A. (2018). Estrogen, angiogenesis, immunity and cell metabolism: Solving the puzzle. Int. J. Mol. Sci..

[70] Guo Y., Ren C., Huang W., Yang W., Bao Y. (2022). Oncogenic ACSM1 in prostate cancer is through metabolic and extracellular matrix-receptor interaction signaling pathways. Am. J. Cancer Res..

[71] Zhang Q.-J., Li D.-Z., Lin B.-Y., Geng L., Yang Z., Zheng S.-S. (2022). SNHG16 promotes hepatocellular carcinoma development via activating ECM receptor interaction pathway. Hepatob. Pancreat. Dis. Int..

[72] Mishra Y.G., Manavathi B. (2021). Focal adhesion dynamics in cellular function and disease. Cell Signal..

[73] Machackova T., Vychytilova-Faltejskova P., Souckova K., Trachtova K., Brchnelova D., Svoboda M., Kiss I., Prochazka V., Kala Z., Slaby O. (2020). MiR-215-5p reduces liver metastasis in an experimental model of colorectal cancer through regulation of ECM-receptor interactions and focal adhesion. Cancers.

[74] Han R., Gu S., Zhang Y., Luo A., Jing X., Zhao L., Zhao X., Zhang L. (2018). Estrogen promotes progression of hormone-dependent breast cancer through CCL2-CCR2 axis by upregulation of Twist via PI3K/AKT/NF-κB signaling. Sci. Rep..

[75] Pei H., Wang W., Zhao D., Su H., Su G., Zhao Z. (2019). G protein-coupled estrogen receptor 1 inhibits angiotensin II-induced cardiomyocyte hypertrophy via the regulation of PI3K-Akt-mTOR signalling and autophagy. Int. J. Biol. Sci..

[76] National Research Council (2012). Nutrient Requirements of Swine.

[77] Zhang Q., Huang L., Leng B., Li Y., Jiao N., Jiang S., Yang W., Yuan X. (2021). Zearalenone Affect the Intestinal Villi Associated with the Distribution and the Expression of Ghrelin and Proliferating Cell Nuclear Antigen in Weaned Gilts. Toxins.

[78] Zhang P., Jing C., Liang M., Jiang S., Huang L., Jiao N., Li Y., Yang W. (2021). Zearalenone Exposure Triggered Cecal Physical Barrier Injury through the TGF-β1/Smads Signaling Pathway in Weaned Piglets. Toxins.

